# Critical role of PPARγ in myeloid-derived suppressor cell-stimulated cancer cell proliferation and metastasis

**DOI:** 10.18632/oncotarget.6414

**Published:** 2015-11-27

**Authors:** Ting Zhao, Hong Du, Janice S. Blum, Cong Yan

**Affiliations:** ^1^ Department of Pathology and Laboratory Medicine, Indiana University School of Medicine, Indianapolis, IN 46202, USA; ^2^ IU Simon Cancer Center, Indiana University School of Medicine, Indianapolis, IN 46202, USA; ^3^ Department of Microbiology and Immunology, Indiana University School of Medicine, Indianapolis, IN 46202, USA

**Keywords:** lysosomal acid lipase, lipid metabolic signaling, myeloid-derived suppressor cells, peroxisome proliferator-activated receptor-γ, tumor growth and metastasis

## Abstract

Lysosomal acid lipase (LAL) is a key enzyme controlling neutral lipid metabolic signaling in myeloid-derived suppressor cells (MDSCs). MDSCs from LAL-deficient (*lal*^−/−^) mice directly stimulate cancer cell proliferation. PPARγ ligand treatment inhibited *lal*^−/−^ MDSCs stimulation of tumor cell growth and metastasis *in vivo*, and tumor cell proliferation and migration *in vitro*. In addition, PPARγ ligand treatment impaired *lal*^−/−^ MDSCs transendothelial migration, and differentiation from lineage-negative cells. The corrective effects of PPARγ ligand on *lal*^−/−^ MDSCs functions were mediated by regulating the mammalian target of rapamycin (mTOR) pathway, and subsequently blocking MDSCs ROS overproduction. Furthermore, in the myeloid-specific dominant-negative PPARγ (dnPPARγ) overexpression bitransgenic mouse model, tumor growth and metastasis were enhanced, and MDSCs from these mice stimulated tumor cell proliferation and migration. MDSCs with dnPPARγ overexpression showed increased transendothelial migration, overactivation of the mTOR pathway, and ROS overproduction. These results indicate that PPARγ plays a critical role in neutral lipid metabolic signaling controlled by LAL, which provides a mechanistic basis for clinically targeting MDSCs to reduce the risk of cancer proliferation, growth and metastasis.

## INTRODUCTION

Lysosomal acid lipase (LAL) hydrolyzes cholesteryl esters and triglycerides in the lysosome of cells to generate free fatty acids and cholesterol. Genetic ablation of the *lal* gene in mice results in a systemic increase of myeloid lineage cells, causing severe inflammation in multiple organs [1, 2]. Myeloid-derived suppressor cells (MDSCs), with co-expression of myeloid-cell lineage differentiation antigen Gr-1 and CD11b in mice, are a heterogeneous population of immature myeloid cells at different stages of differentiation [3]. We previously reported that the neutral lipid metabolic pathway controlled by LAL plays a critical role in the development and homeostasis of MDSCs, and demonstrated that LAL deficiency leads to the infiltration and accumulation of MDSCs in various organs of the mice, such as the lung, spleen, thymus, liver and small intestine [1, 4]. In addition to immune suppressive function, our recent studies showed that LAL-deficient (*lal*^−/−^) MDSCs possess direct tumor stimulatory function [5]. By Affymetrix GeneChip microarray, we have identified that many important gene pathways are involved in the dysfunctions of *lal*^−/−^ MDSCs, notably among which, peroxisome proliferator-activated receptor-γ (PPARγ)-regulated gene expression is significantly altered [6].

PPARγ, a member of the nuclear receptor superfamily, serves as the receptor of free fatty acid derived compounds which arise downstream of LAL enzymatic action. After binding to these ligands, PPARγ plays an important role in limiting inflammation in various tissues by suppressing the expression of inflammatory cytokines [7, 8]. Overexpression of pro-inflammatory molecules (e.g., apoptosis inhibitor 6 and matrix metalloproteinase 12) that are negatively regulated by PPARγ has been reported to induce chronic inflammation and spontaneous tumor formation [9–13]. Our previous study has suggested that LAL deficiency causes inactivation of PPARγ by blocking ligand generation, which in turn promotes pulmonary inflammation and pathogenesis [14]. Furthermore, by using the dominant-negative PPARγ (dnPPARγ) myeloid-specific overexpression bitransgenic mouse model, we found that PPARγ plays a key role in controlling pro-inflammatory cytokine synthesis, MDSC expansion, immunosuppression, and the development of cancer [15].

Since LAL downstream metabolic derivatives serve as hormonal ligands for PPARγ, the current study examined if PPARγ plays an important role in LAL-mediated functions in MDSCs. Here, the corrective effects of the PPARγ ligand 9-hydroxyoctadecadienoic acid (9-HODE) on the neutral lipid metabolic signaling controlled by LAL are examined, including effects on the development and function of MDSCs, MDSCs transendothelial migration, tumor cell proliferation and metastasis. The results demonstrate that 9-HODE treatment corrected the defects in *lal*^−/−^ MDSCs via effecting the mammalian target of rapamycin (mTOR) pathway and by inhibiting overproduction of reactive oxygen species (ROS). These findings provide novel mechanistic insights into the linkage between lipid metabolic signaling and PPARγ in MDSC dysfunction. Importantly, these studies reveal the critical role of LAL and PPARγ in checking MDSC functions and the potential as a therapeutic target to modulate tumor growth and spread associated with MDSCs.

## RESULTS

### PPARγ ligand impaired *lal*^−/−^ Ly6G^+^ cell stimulation of tumor growth and metastasis *in vivo*

**Figure 1:**
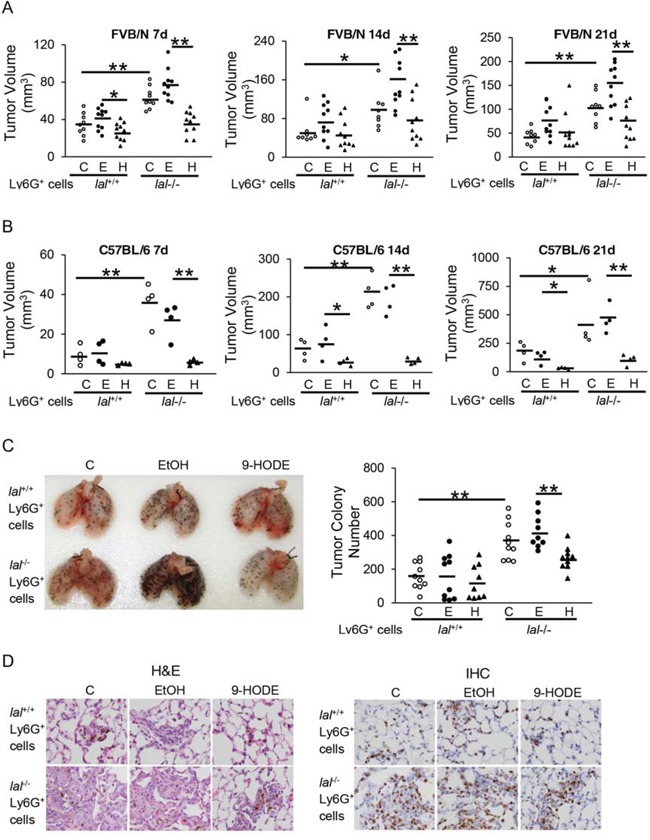
PPARγ ligand reverses *lal*^−/−^ MDSCs stimulation on tumor growth and metastasis *in vivo* **A.** Ly6G^+^ cells from *lal*^+/+^ or *lal*^−/−^ FVB/N mice were pre-treated with ethanol (E) or 20 μmol/L 9-HODE (H) or without treatment (C) for 24 h. Pre-treated Ly6G^+^ cells (6 × 10^5^) and B16 melanoma cells (2 × 10^5^, without any treatment) were mixed, and co-injected subcutaneously into the flank region of 3-month old *lal*^+/+^ FVB/N mice. *n* = 8∼10. **B.** Pre-treated C57BL/6 Ly6G^+^ cells (6 × 10^5^) and B16 melanoma cells (2 × 10^5^) were co-injected subcutaneously into the flank region of 3-month old *lal*^+/+^ C57BL/6 mice. *n* = 4. Tumor volume (in cubic millimeters) were measured and statistically analyzed at 7, 14, and 21 days post-injection. For statistical analyses, data were expressed as mean ± SD. **P < 0.01, *P < 0.05. **C.** Pre-treated Ly6G^+^ cells (2 × 10^6^) and B16 melanoma cells (5 × 10^5^, without any treatment) were intravenously co-injected into *lal*^+/+^ mice for 2 weeks. Representative lungs and quantitative analysis of the melanoma colony numbers in the lungs are shown. Data were expressed as mean ± SD; *n* = 9∼10. **P < 0.01. **D.** Representative H&E staining and IHC staining with Ki67 antibody of the lungs with metastasized melanoma are shown. Original magnification, × 400.

PPARγ inactivation has previously been reported to cause inflammation-triggered cell growth and emphysema in *lal*^−/−^ mice, and treatment with the PPARγ ligand 9-HODE significantly rescued *lal*^−/−^ pulmonary inflammation and aberrant gene expression [14]. *lal*^−/−^ Ly6G^+^ MDSCs have recently been found to play a role in stimulating tumor growth and metastasis [5]. Based on the literature and our own experience, the wild type bone marrow is comprised of ∼50% CD11b^+^Ly6G^+^ myeloid precursor cells (with very low immunosuppressive function) and very few CD11b^+^ or Ly6G^+^ single cells. On the other hand, the *lal*^−/−^ bone marrow is comprised of 70% CD11b^+^Ly6G^+^ cells (with very strong immunosuppression and cancer cell stimulation potential). These *lal*^−/−^ bone marrow cells do not further differentiate into more mature myeloid cells as wild type cells do [1]. To see whether PPARγ inactivation within *lal*^−/−^ Ly6G^+^ MDSCs contributes to their ability to stimulate tumor cells, freshly isolated bone marrow-derived *lal*^+/+^ or *lal*^−/−^ Ly6G^+^ cells were pre-treated with 9-HODE or the vehicle, ethanol for 24 h. In *lal*^−/−^ mice, since almost all Ly6G^+^ cells are positive for CD11b, a Ly6G-specific antibody was used for purification of Ly6G^+^CD11b^+^ cells. To examine tumor growth potential *in vivo*, pre-treated or untreated Ly6G^+^ cells were mixed with untreated B16 melanoma cells, and then co-injected subcutaneously into *lal*^+/+^ mice. One week after the injection, subcutaneous tumors detected in the *lal*^−/−^ Ly6G^+^ cell-injected mice were significantly larger (tumor volume = 63.2 ± 11.7 mm^3^) than those tumors in *lal*^+/+^ Ly6G^+^ cell-injected mice (tumor volume = 34.6 ± 11.9 mm^3^, *p* < 0.01). However, the tumors from 9-HODE-treated *lal*^−/−^ Ly6G^+^ cell-injected mice (tumor volume = 36.3 ± 12.4 mm^3^) were significantly smaller when compared with those developed in ethanol-treated *lal*^−/−^ Ly6G^+^ cell-injected mice (tumor volume = 77.6 ± 16.4 mm^3^, *p* < 0.01) (Figure 1A). The similar effect of 9-HODE treatment on *lal*^−/−^ ly6G^+^ cells to tumor size was also observed at 14 and 21 days post-injection (Figure 1A). Moreover, when B16 melanoma cells were co-injected with C57BL/6 Ly6G^+^ cells into C57BL/6 mice, similar results were observed that the tumors from 9-HODE-treated *lal*^−/−^ Ly6G^+^ cell-injected mice were significantly smaller than those developed in ethanol-treated *lal*^−/−^ Ly6G^+^ cell-injected mice at 7, 14 and 21 days post-injection (Figure 1B). As predicted, B16 melanoma tumor grew larger in C57BL/6 than that in FVB/N mice at 14 and 21 days post-injection.

Next, the pre-treated Ly6G^+^ cells and B16 melanoma cells were injected into the tail veins of *lal*^+/+^ recipient mice to detect metastatic potential. Two weeks after injection, less B16 melanoma colonies were observed in the lungs of *lal*^+/+^ mice that received 9-HODE-treated *lal*^−/−^ Ly6G^+^ and B16 cell co-injection than those received ethanol-treated *lal*^−/−^ Ly6G^+^ and B16 cell co-injection (Figure 1C). 9-HODE treatment of *lal*^+/+^ ly6G^+^ cells did not affect B16 melanoma colonization in the lung (Figure 1C). Sections of the lungs showed less neoplastic cells by H&E staining and less Ki67 positive cells by IHC staining (Figure 1D). These observations suggest that ligand-induced activation of the PPARγ pathway in *lal*^−/−^ Ly6G^+^ MDSCs impaired the ability of these myeloid cells to stimulate tumor growth and metastasis.

### PPARγ ligand inhibited *lal*^−/−^ Ly6G^+^ MDSCs stimulation of tumor proliferation and migration *in vitro*

**Figure 2:**
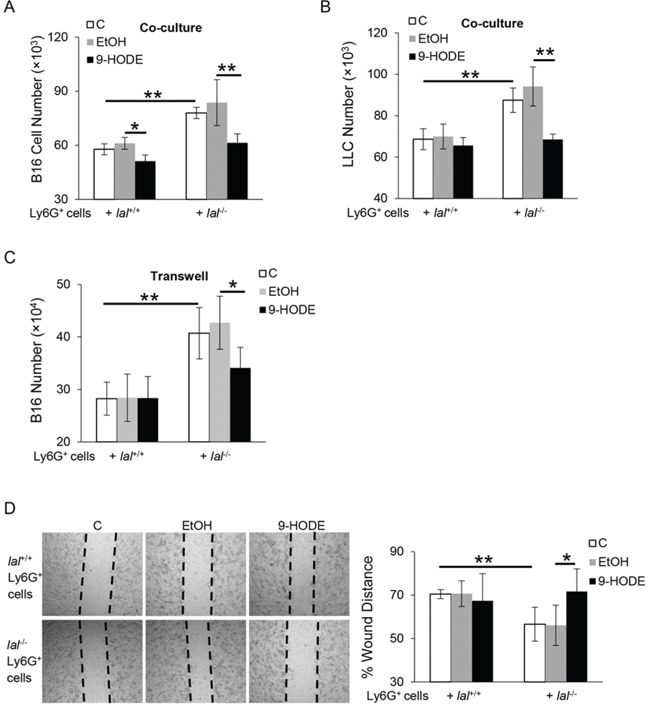
PPARγ ligand inhibits *lal*^−/−^ MDSCs stimulation on tumor proliferation and migration *in vitro* Ly6G^+^ cells from *lal*^+/+^ or *lal*^−/−^ mice were pre-treated with ethanol (EtOH) or 20 μmol/L 9-HODE for 24 h. **A.** Pre-treated Ly6G^+^ cells (5 × 10^5^) were co-cultured with B16 melanoma cells (5 × 10^3^) *in vitro* for 72 h, and numbers of B16 melanoma cells were counted. *n* = 4∼5. **B.** Pre-treated Ly6G^+^ cells (5 × 10^5^) were co-cultured with LLC cells (1 × 10^4^) *in vitro* for 72 h, and numbers of LLC cells were counted. *n* = 4∼5. **C.** To see the effect of Ly6G^+^ cell-secreted cytokines on B16 melanoma cell proliferation, pre-treated Ly6G^+^ cells (1 × 10^6^) were seeded into the upper chamber of transwells, in which B16 melanoma cells (2 × 10^4^) were seeded in the lower chamber. After 72 h, the number of B16 melanoma cells was counted. *n* = 5. **D.** Left: *in vitro* migration of B16 melanoma cells with pre-treated Ly6G^+^ cells at 24 h after co-culture in the presence of mitomycin C. The dotted lines define the areas lacking cells. Right: Quantification of distance from one end of the wound area to the other end. Data were normalized to B16 melanoma cells co-cultured with control *lal*^+/+^ Ly6G^+^ cells at 0 h. Original magnification, × 40. *n* = 5. For statistical analyses, data were expressed as mean ± SD; **P < 0.01, *P < 0.05.

The inhibitory effects of the PPARγ ligand on *lal*^−/−^ Ly6G^+^ MDSCs stimulation of tumor growth were further examined by *in vitro* co-culture experiments. Ligand or vehicle pre-treated *lal*^+/+^ or *lal*^−/−^ Ly6G^+^ cells were co-cultured with B16 melanoma cells for 72 h. As shown in Figure 2A, 9-HODE treatment of *lal*^−/−^ Ly6G^+^ cells significantly decreased proliferation of B16 melanoma cells upon co-culture, compared with that of ethanol-treated *lal*^−/−^ Ly6G^+^ cells. When 9-HODE-treated *lal*^−/−^ Ly6G^+^ cells were co-cultured with Lewis lung cancer (LLC) cells, reduced proliferation of LLC cells was also observed (Figure 2B). Taken together, these results suggest that activation of the PPARγ pathway in *lal*^−/−^ Ly6G^+^ cells impaired the capacity of these myeloid cells to stimulate tumor cell proliferation.

Cytokines secreted by *lal*^−/−^ Ly6G^+^ MDSCs have been reported to be responsible for mediating their stimulatory effects on cancer cell proliferation [5]. To examine whether 9-HODE treatment has an effect on cytokine-mediated Ly6G^+^ MDSCs stimulation on cancer cell proliferation, transwell studies were performed with 9-HODE pre-treated Ly6G^+^ cells seeded in the upper chamber and melanoma cells seeded in the lower chamber. After 72 h co-culture, the number of B16 melanoma cells that were co-cultured with 9-HODE pre-treated *lal*^−/−^ Ly6G^+^ cells was significantly less (Figure 2C), suggesting the ability of *lal*^−/−^ Ly6G^+^ cells to promote melanoma cell proliferation was impaired by PPARγ ligand treatment.

Because cell migration contributes to metastasis, *in vitro* tumor cell migration assay was analyzed to determine whether PPARγ ligand treatment of *lal*^−/−^ Ly6G^+^ cells influences B16 melanoma cell migration. Melanoma cells were treated with mitomycin C to eliminate the potential effects of cell proliferation in these assays. As shown in Figure 2D, 24 h after co-culture with *lal*^−/−^ Ly6G^+^ cells, B16 melanoma cells migrated more efficiently into the area of an artificial wound area compared with those tumor cells co-cultured with *lal*^+/+^ Ly6G^+^ cells. However, delayed migration towards the scratch was observed in 9-HODE pre-treated *lal*^−/−^ Ly6G^+^ cells, as revealed by a significant increase in the span of the wounded area. These results also suggest that activation of the PPARγ pathway in *lal*^−/−^ Ly6G^+^ cells impaired the stimulatory effects of these MSDCs on B16 melanoma cell migration *in vitro*.

### PPARγ ligand decreased *lal*^−/−^ MDSC transendothelial migration capability and differentiation from *lal*^−/−^ Lin^−^ cells

**Figure 3:**
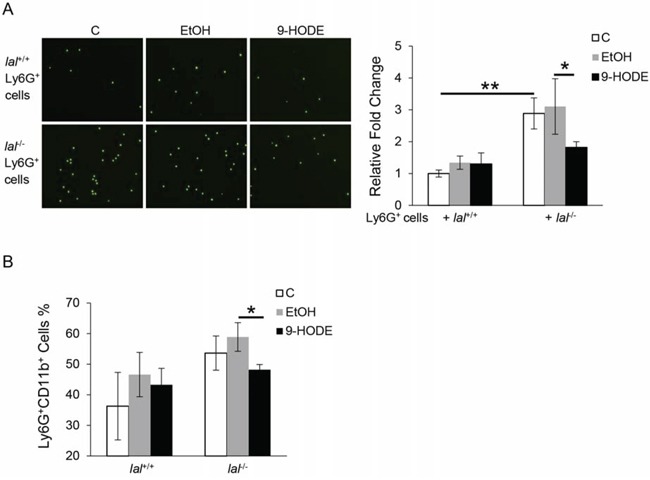
PPARγ ligand decreases *lal*^−/−^ MDSCs transendothelial migration capacity and differentiation from *lal*^−/−^ Lin- cells **A.** Transwell assay was performed to determine MDSCs transmigration across the endothelial monolayer. Ly6G^+^ cells from *lal*^+/+^ or *lal*^−/−^ mice were pre-treated with ethanol (EtOH) or 20 μmol/L 9-HODE for 48 h, and then labeled with CMFDA and seeded onto the endothelial monolayer at a density of 2 × 10^4^ cells/well. Four hours after seeding Ly6G^+^ cells on the EC monolayer, the number of Ly6G^+^ cells that have migrated to the lower chamber was counted. **B.** Statistical analysis of Ly6G^+^CD11b^+^ cells from Lin^−^ cells that were treated with ethanol (EtOH) or 10 μmol/L 9-HODE for 5 days by flow cytometry. Data were expressed as mean ± SD; *n* = 3∼4. **P < 0.01, *P < 0.05.

Besides effects on tumor growth and metastasis, *lal*^−/−^ Ly6G^+^ MDSCs displayed increased transendothelial migration capability [16], which likely results in the severe infiltration of MDSCs in multiple organs of *lal*^−/−^ mice. To test whether PPARγ inactivation in *lal*^−/−^ Ly6G^+^ cells plays a role in their increased transendothelial migration, transwell assays were performed with 9-HODE pre-treated CMFDA-labeled Ly6G^+^ cells seeded onto an endothelial monolayer in the upper chamber of the plates. Four hours later, the number of Ly6G^+^ cells that had migrated through to the lower chamber was determined. As shown in Figure 3A, there were less Ly6G^+^ cells in the lower chamber when *lal*^−/−^ Ly6G^+^ cells were treated with 9-HODE compared with these cells treated with ethanol, suggesting that the PPARγ pathway is involved in Ly6G^+^ cell endothelial transmigration capability.

Abnormal expansion of MDSCs was also observed in *lal*^−/−^ mice, which was due to increased differentiation from Lin^−^ cells [1]. PPARγ is known to be expressed in bone marrow progenitor cells and play a critical role in mesenchymal stem cell differentiation and adipogenesis [17–19]. To test the role of PPARγ in this process, bone marrow-derived Lin^−^ cells from *lal*^+/+^ and *lal*^−/−^ mice were isolated and treated with 9-HODE or ethanol. After 5 days of incubation, fewer Ly6G^+^CD11b^+^ cells were derived from 9-HODE-treated *lal*^−/−^ Lin^−^ cells compared with those with ethanol treatment (Figure 3B), suggesting that activation of the PPARγ pathway by 9-HODE prevented these Lin^−^ cells from differentiating into MDSCs.

### PPARγ ligand down-regulated mTOR pathway activation in *lal*^−/−^ Ly6G^+^CD11b^+^ cells

**Figure 4:**
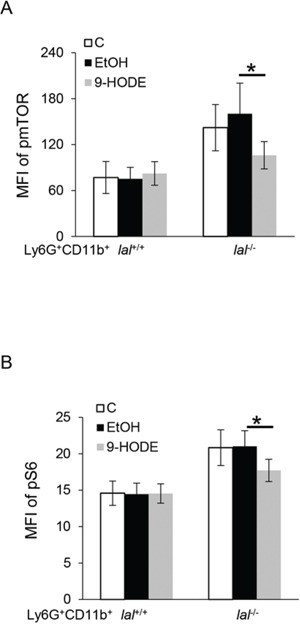
PPARγ ligand down-regulates the overactivation of the mTOR pathway in *lal*^−/−^ MDSCs Bone marrow cells from *lal*^+/+^ or *lal*^−/−^ mice were treated with ethanol (EtOH) or 20 μmol/L 9-HODE for 2 h. **A.** 9-HODE decreased phosphorylation of mTOR in gated *lal*^−/−^ Ly6G^+^CD11b^+^ cells. **B.** 9-HODE decreased phosphorylation of S6 in gated *lal*^−/−^ Ly6G^+^CD11b^+^ cells. Statistical analysis of mean fluorescent intensity (MFI) by flow cytometry is shown. Data were expressed as mean ± SD; *n* = 7. *P < 0.05.

We have previously reported that the tumor-promoting function of *lal*^−/−^ MDSCs is mediated, at least in part, through enhanced activation of the mTOR pathway [5], and that the mTOR pathway is involved in the differentiation of Lin^−^ cells into Ly6G^+^CD11b^+^ cells [20]. To test whether PPARγ has an effect on the mTOR pathway, bone marrow cells from *lal*^+/+^ and *lal*^−/−^ mice were treated with 9-HODE or ethanol. After 2 h of incubation, the expression levels of phosphorylated mTOR (pmTOR) and phosphorylated S6 (pS6) in gated Ly6G^+^CD11b^+^ cells were measured by flow cytometry analysis. As shown in Figure 4, the increased levels of pmTOR and pS6 in *lal*^−/−^ Ly6G^+^CD11b^+^ cells were not observed in cells following PPARγ ligand treatment. These results suggest that ligand-induced activation of the PPARγ pathway in *lal*^−/−^ Ly6G^+^CD11b^+^ cells by 9-HODE downregulated mTOR pathway activation.

### PPARγ ligand reversed damaged mitochondrial membrane potential and suppressed ROS production in *lal*^−/−^ Ly6G^+^CD11b^+^ cells

**Figure 5:**
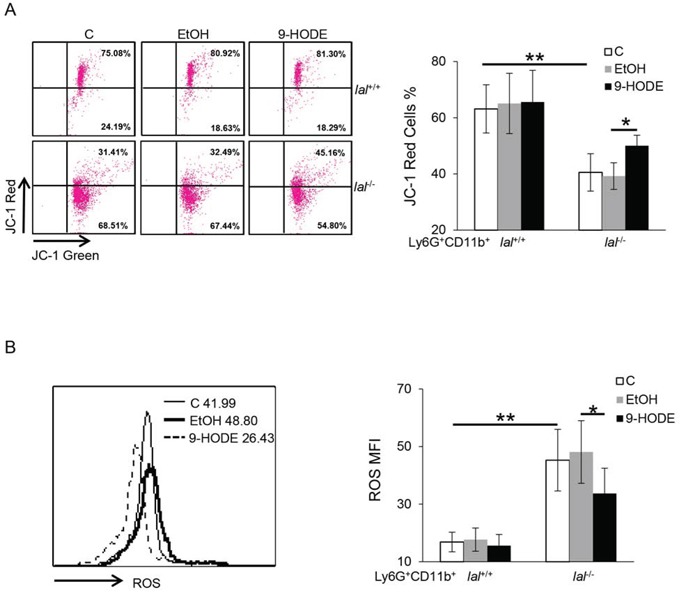
PPARγ ligand reverses the damaged mitochondrial membrane potential and suppresses ROS production in *lal*^−/−^ MDSCs Bone marrow cells from *lal*^+/+^ or *lal*^−/−^ mice were treated with ethanol (EtOH) or 20 μmol/L 9-HODE for 48 h. **A.** 9-HODE increased the mitochondrial membrane potential in gated *lal*^−/−^ Ly6G^+^CD11b^+^ cells. Left: Representative dot plot analysis of the JC-1 red and JC-1 green profiles by flow cytometry. Right: Statistical analysis of the mitochondrial membrane potential in Ly6G^+^CD11b^+^ cells. **B.** 9-HODE decreased ROS production in *lal*^−/−^ Ly6G^+^CD11b^+^ cells. Left: Representative analysis of MFI by flow cytometry. Right: Statistical analysis of MFI in Ly6G^+^CD11b^+^ cells. Data were expressed as mean ± SD; *n* = 5∼6. **P < 0.01, *P < 0.05.

ROS is an important mediator for MDSCs functions, and its increase is often associated with mitochondrial damage. In *lal*^−/−^ MDSCs, both damaged mitochondrial function and ROS overproduction have been observed, and inhibition of the mTOR pathway decreased the ROS levels and abnormal mitochondrial membrane potential in *lal*^−/−^ MDSCs [6]. To see whether PPARγ ligand treatment corrects these defects, bone marrow cells were treated with 9-HODE or ethanol, and ROS levels and mitochondrial membrane potentials were measured by flow cytometry analysis. As demonstrated in Figure 5A, the impaired mitochondrial membrane potential in *lal*^−/−^ Ly6G^+^ CD11b^+^ cells was partially recovered with 9-HODE treatment compared with that in ethanol-treated cells. In addition, 9-HODE treatment suppressed the increased ROS production in *lal*^−/−^ Ly6G^+^CD11b^+^ cells (Figure 5B). These results suggest that ROS overproduction and damaged mitochondrial membrane potential associated with *lal*^−/−^ MDSCs can be corrected by PPARγ ligand treatment.

### Overexpression of dnPPARγ in myeloid cells facilitated tumor growth and metastasis *in vivo*, and tumor proliferation and migration *in vitro*

**Figure 6:**
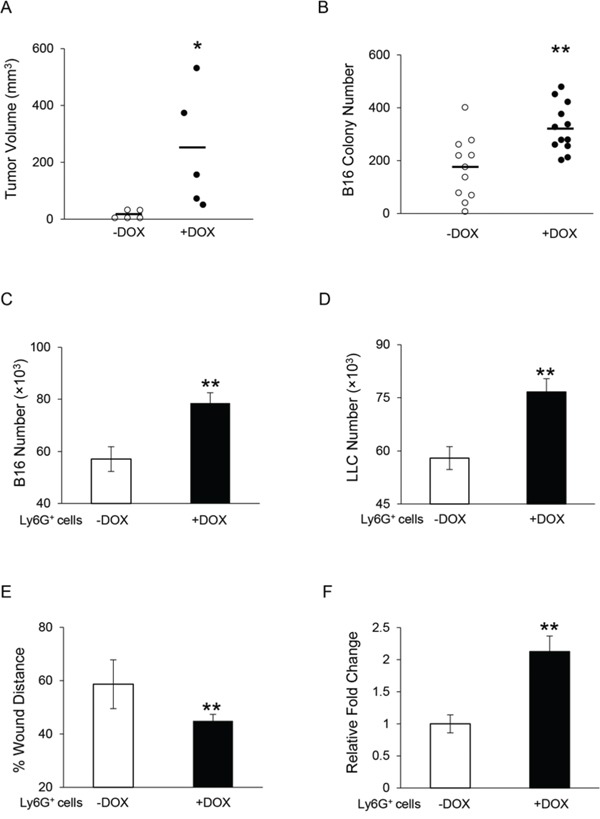
Overexpression of dnPPARγ in myeloid cells facilitates tumor growth and metastasis *in vivo*, and tumor proliferation and migration *in vitro* **A.** Statistical analysis of tumor volume (in cubic millimeters) at 4 weeks after B16 melanoma cells (2 × 10^5^) were subcutaneously injected into doxycycline-treated or untreated c-fmsrtTA/(tetO)7-dnPPARγ bitransgenic mice. *n* = 5. *P < 0.05. **B.** Quantitative analysis of metastasized B16 melanoma colonies in the lungs of doxycycline-treated or untreated bitransgenic mice with intravenous injection of 5 × 10^5^ B16 melanoma cells for 2 weeks. *n* = 11∼12. **P < 0.01. **C.** B16 melanoma cells (5 × 10^3^) were co-cultured with Ly6G^+^ cells (5 × 10^5^) from doxycycline-treated or untreated bitransgenic mice *in vitro* for 72 h, and numbers of B16 melanoma cells were counted. **D.** LLC cells (1 × 10^4^) were co-cultured with doxycycline-treated or untreated Ly6G^+^ cells (5 × 10^5^) *in vitro* for 72 h, and the numbers of LLC cells were counted. **E.** *In vitro* migration of B16 melanoma cells with doxycycline-treated or untreated Ly6G^+^ cells at 24 h after co-culture in the presence of mitomycin C. Data were normalized to B16 melanoma cells co-cultured with untreated Ly6G^+^ cells at 0 h. **F.** Ly6G^+^ cell transendothelial migration was determined. Data are normalized to untreated Ly6G^+^ cells. In the above experiments (C-F), data were expressed as mean ± SD; *n* = 4. **P < 0.01.

To further confirm the critical role of PPARγ in myeloid-lineage cells, a doxycycline-inducible c-fms-rtTA/(tetO)_7_-CMV-dnPPARγ bitransgenic mouse model was used, in which a dominant negative PPARγ (dnPPARγ) was overexpressed in myeloid cells under the control of the c-fms promoter [15]. As we published before when the endogenous PPARγ signaling pathway was inhibited by overexpression of dnPPARγ in myeloid cells, the MDSCs level increased in bone marrow, spleen, blood and lung [15]. Here we assess whether the disruption of PPARγ function by expression of dnPPARγ in myeloid cells has similar effect on tumor cell growth and metastasis *in vivo* and tumor cell proliferation and migration *in vitro*. In tumor growth assessment, B16 melanoma cells were subcutaneously injected into the flank region of the bitransgenic mice. Figure 6A showed that the tumor volume from doxycycline-treated bitransgenic mice was significantly increased compared with those in untreated mice at 4 weeks post-injection. For the tumor metastasis potential, statistical analysis revealed that two weeks after intravenous injection of B16 melanoma cells, the doxycycline-treated bitransgenic mice showed increased number of melanoma colonies in the lungs compared with untreated mice (Figure 6B). These results suggest that PPARγ inactivation in myeloid cells contributed to the increased tumor growth and metastasis.

When bone marrow Ly6G^+^ cells from doxycycline-treated bitransgenic mice were co-cultured with B16 melanoma cells *in vitro*, increased proliferation of B16 melanoma cells was observed in comparison with those cells from untreated bitransgenic mice (Figure 6C). Similarly, proliferation of LLC was significantly increased after co-cultured with bone marrow Ly6G^+^ cells from doxycycline-treated bitransgenic mice (Figure 6D). Furthermore, the *in vitro* wound healing assay showed accelerated migration towards the scratch in B16 melanoma cells co-cultured with bone marrow Ly6G^+^ cells from doxycycline-treated bitransgenic mice 24 h after creating the scratch, with a significant decrease of distance in the wounding area (Figure 6E). In addition, the transendothelial migration capability of Ly6G^+^ cells from doxycycline-treated bitransgenic mice was obviously increased as shown in Figure 6F. Taken together, these results indicate that PPARγ inactivation in Ly6G^+^ cells facilitated their transendothelial migration, and stimulation of tumor cell proliferation and migration.

### Overexpression of dnPPARγ in myeloid cells overactivated the mTOR pathway, increased ROS production and impaired maintenance of mitochondrial membrane potential

**Figure 7:**
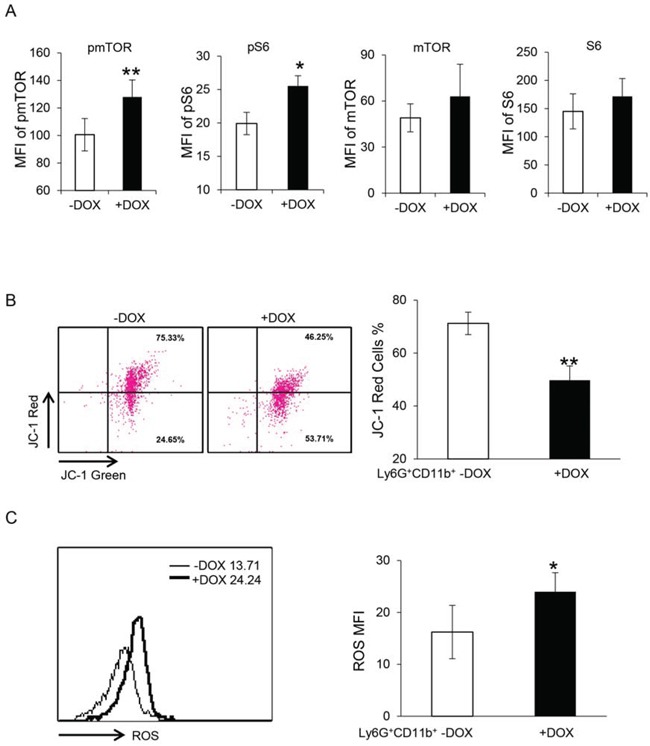
Overexpression of dnPPARγ in myeloid cells overactivates the mTOR pathway, increases ROS production and impairs mitochondrial membrane potential **A.** The mTOR pathway was overactivated in doxycycline-treated bone marrow Ly6G^+^CD11b^+^ cells. Statistical analysis of MFI by flow cytometry is shown. Data were expressed as mean ± SD; *n* = 4∼5. **P < 0.01, *P < 0.05. **B.** The mitochondrial membrane potential was impaired in doxycycline-treated bone marrow Ly6G^+^CD11b^+^ cells. Left: Representative dot plot analysis of the JC-1 red and JC-1 green profiles by flow cytometry. Right: Statistical analysis of the mitochondrial membrane potential in Ly6G^+^CD11b^+^ cells. **C.** ROS production was increased in doxycycline-treated Ly6G^+^CD11b^+^ cells. Left: Representative analysis of MFI by flow cytometry. Right: Statistical analysis of MFI in Ly6G^+^CD11b^+^ cells. For statistical analyses, data were expressed as mean ± SD; *n* = 5. **P < 0.01, *P < 0.05.

To explore the potential mechanisms underlying the dysfunctions of MDSCs from doxycycline-treated dnPPARγ bitransgenic mice, changes in the mTOR pathway were explored. As determined above using PPARγ ligands, the pathogenic function of MDSCs could be linked to mTOR activation in *lal*^−/−^ MDSCs. Results showed that the phosphorylation levels of mTOR and S6 in gated doxycycline-treated Ly6G^+^CD11b^+^ cells were increased significantly compared with those of untreated Ly6G^+^CD11b^+^ cells, with no statistically significant change of mTOR and S6 protein levels (Figure 7A). As a consequence, the mitochondrial membrane potential in doxycycline-treated Ly6G^+^CD11b^+^ cells was impaired (Figure 7B). In addition, the ROS production in doxycycline-treated Ly6G^+^CD11b^+^ cells was significantly increased compared with untreated Ly6G^+^CD11b^+^ cells (Figure 7C). These results support that the PPARγ pathway regulates MDSCs functions by modulating mTOR, ROS production and mitochondrial membrane potential.

## DISCUSSION

Activating PPARγ can prevent cancer development and spread in tissues such as colon, breast, prostate and lung, while ligand-induced activation of PPARγ in cancer cell lines is associated with the induction of cell cycle arrest, the increased expression of mRNAs and proteins required for terminal differentiation, as well as changes in cell morphology that are consistent with a differentiated phenotype [21]. Yet understanding pathways by which PPARγ contributes to the spread and progression of cancer *in vivo,* including its effects on immune cells is less well understood. LAL is a key enzyme which functions in the metabolism of neutral lipids, and its role in inflammation has been widely studied [1, 4, 20, 22]. Genetic ablation of the *lal* gene in mice results in a systemic increases in MDSCs and decreases in T cell populations, causing severe inflammation and pathogenesis in multiple organs [1, 23]. LAL deficiency causes inactivation of PPARγ by blocking PPARγ ligand synthesis [14]. The PPARγ signaling pathway has recently been reported to play a key role in controlling MDSC expansion and T cell proliferation [15]. Here, 9-HODE, a PPARγ ligand [24], reversed the increased MDSC expansion (Figure 3B) and decreased T cell numbers in *lal*^−/−^ mice (data not shown), suggesting that PPARγ signaling is critical in regulating LAL-mediated metabolic pathways central to immune suppression [15]. Interestingly and importantly, our previous [5] and present studies have shown that *lal*^−/−^ MDSCs were able to also overcome potent immune rejection and destruction of tumors in allogeneic mouse models.

In addition to immunosuppression, we have shown that *lal*^−/−^ MDSCs have a second potent effect on tumors, by directly stimulating tumor cell proliferation, growth and metastasis [5]. B16 melanoma cells grew quickly and metastasized massively in allogeneic *lal*^−/−^ mice, yet this effect was suppressed in allogeneic *lal*^+/+^ mice where immune destruction of the tumor took place. However, *lal*^−/−^ MDSCs facilitated melanoma cells' growth and metastasis in allogeneic *lal*^+/+^ mice [5]. In our preliminary study, B16 melanoma cells' metastasis was delayed in *lal*^−/−^ mice pre-treated with PPARγ ligand 9-HODE. In the present study, activation of the PPARγ pathway in *lal*^−/−^ MDSCs with its ligand 9-HODE not only impaired their stimulatory effects on *in vivo* tumor growth and metastasis (Figure 1), but also significantly retarded the ability of these MSDCs to block *in vitro* tumor cell proliferation and migration (Figure 2). We have reported that cytokines, (especially TNFα) secreted by *lal*^−/−^ Ly6G^+^ cells are, at least in part, responsible for mediating stimulatory effects on cancer cells [5]. In the present study, 9-HODE inhibited the cytokine-mediated stimulatory effects of *lal*^−/−^ MDSCs on melanoma cell proliferation (Figure 2C). Therefore, PPARγ inactivation in *lal*^−/−^ MDSCs represents a major mechanism underlying the stimulatory effects of MSDCs on cancer cell proliferation and metastasis.

One major manifestation during LAL deficiency is systemic MDSCs expansion and dysfunction in multiple organs of the mice [1, 2, 14, 22, 25], which arises from dysregulated production of hematopoietic myeloid progenitor cells in the bone marrow [1]. When *lal*^−/−^ Lin^−^ cells were treated with 9-HODE to activate the PPARγ pathway, reduced differentiation of Lin^−^ cells into Ly6G^+^CD11b^+^ MDSCs was observed (Figure 3B). Kaipainen *et al* showed that PPARγ ligands inhibit primary tumor growth and metastasis by targeting endothelial cells to inhibit angiogenesis [26]. Abnormal accumulation of MDSCs in the lungs of *lal*^−/−^ mice has been linked to both endothelial cells (ECs) and MDSCs [16]. Activation of the PPARγ pathway in *lal*^−/−^ MDSCs with 9-HODE decreased the transendothelial migration of MDSCs through the EC monolayer (Figure 3A). These results collectively indicate that activation of the PPARγ pathway corrects *lal*^−/−^ MDSCs dysfunction and abnormal expansion during LAL deficiency.

In addition to inhibition of the PPARγ pathway, enhanced activation of the mTOR pathway was associated with *lal*^−/−^ MDSC dysfunction as detected by Affymetrix GeneChip microarray and Ingenuity analyses [6]. Thus, multiple pathways may contribute to regulate MSDCs functions. Studies have shown that the mTOR pathway regulates PPARγ activation during adipogenesis by targeting the transactivation activity of PPARγ [27, 28]. Interaction between mTOR and PPARγ has been reported before in hepatocytes [29]. Deficiency of PPARγ in chondrocytes resulted in aberrant activation of mTOR signaling pathway [30]. The present study shows that the mTOR pathway in *lal*^−/−^ MDSCs is regulated by PPARγ. Incubation with 9-HODE not only significantly decreased the phosphorylation levels of mTOR and S6, but also reduced the overall levels of mTOR and S6 in *lal*^−/−^ MDSCs (Figure 4). ROS production has been reported to be one mechanism underlying MDSCs functions [3]. In our previous studies, ROS production was increased in *lal*^−/−^ MDSCs with impaired mitochondrial function, which mediated the mTOR-regulated *lal*^−/−^ MDSCs dysfunctions [6, 20]. Activation of the PPARγ pathway in *lal*^−/−^ bone marrow cells with its ligand 9-HODE effectively improved the mitochondrial function and blocked ROS overproduction in *lal*^−/−^ Ly6G^+^CD11b^+^ MDSCs (Figure 5), suggesting that ROS overproduction by *lal*^−/−^ MDSCs is controlled by the PPARγ pathway. Therefore, the mTOR-ROS pathway serves as a potential mechanism to mediate the LAL-PPARγ axis in MDSC dysfunctions. Kittler *et al* recently found that PPARγ inhibits cancer cell proliferation by a metabolic switch, including suppressing pyruvate oxidation and reducing glutathione levels, which results in a marked increase of ROS levels, leading to rapid hypophosphorylation of retinoblastoma protein and cell-cycle rest [31]. Similarly, in a “*lal*^−/−^ MDSCs-like cell line”, we have observed an mTOR-controlled metabolic switch towards increased glycolysis and ROS levels ^30^. In the current study, with PPARγ ligand treatment of *lal*^−/−^ MDSCs we found these cells displayed reduced ROS and were unable to effectively stimulate tumor cell proliferation.

The role of the PPARγ pathway in MDSC functions was further investigated using an established bitransgenic mouse model, in which dnPPARγ was overexpressed in myeloid-lineage cells, resulting in blockade of endogenous PPARγ function [15]. In this mouse model, the function of the receptor of PPARγ pathway is impaired rather than the ligand expression which is perturbed in *lal*^−/−^ mouse model. When melanoma cells were injected subcutaneously into these mice, larger tumor developed in the mice with myeloid-specific dnPPARγ overexpression induced by doxycycline than non-induced bitransgenic mice (Figure 6A). In addition, after intravenous injection of melanoma cells, more melanoma developed in the lungs of mice with myeloid-specific dnPPARγ overexpression (Figure 6B). When MDSCs from the mice with myeloid-specific dnPPARγ overexpression were co-cultured with B16 melanoma cells or LLC cells *in vitro*, cancer cell proliferation was enhanced (Figure 6C and 6D). Moreover, these MDSCs facilitated melanoma cell migration (Figure 6E), possessed increased transmigration through the EC monolayer (Figure 6F), over-activated the mTOR pathway, and impaired mitochondrial function and ROS overproduction (Figure 7), similar to the characteristics observed in *lal*^−/−^ MDSCs, which showed inactivation of the PPARγ pathway.

In conclusion, the PPARγ pathway plays a critical role in metabolic signaling controlled by LAL through regulating the function of MDSCs. The PPARγ pathway served as a novel target to modulate the emergence of MDSCs to reduce the risk of cancer progression and metastasis. It has been extensively reported that PPARγ ligands have a direct inhibitory effect on tumor cells [32–40]. But their effect in MDSCs of the tumor microenviroment is poorly understood, and should be given a special attention. Therefore, PPARγ may impact cancer cell proliferation through both direct and indirect mechanisms including effects on MSDCs. The study outlined here indicates that enhancing PPARγ function in MDSCs should prove to be a highly effective strategy in blocking tumor cell growth and spread even in cases where tumors may not respond directly to PPARγ ligands. Among collection of ligands to PPARγ, those more likely to achieve this therapeutic outcome in MDSCs remain to be tested.

## MATERIALS AND METHODS

### Animals and cell lines

Wild-type (*lal*^+/+^) and *lal*^−/−^ mice of the FVB/N background were bred in house. c-fms-rtTA/(TetO)_7_-CMV-dnPPARγ bitransgenic mice of the FVB/N background is a previously generated bitransgenic mouse model [15]. All scientific protocols involving the use of animals have been approved by the Institutional Animal Care and Use Committee of Indiana University School of Medicine and followed guidelines established by the Panel on Euthanasia of the American Veterinary Medical Association. Animals were housed under Institutional Animal Care and Use Committee-approved conditions in a secured animal facility at Indiana University School of Medicine.

The murine B16 melanoma cell line, Lewis lung carcinoma (LLC) cell line, and murine endothelial cell (SVEC) line (purchased from ATCC, Manassas, VA, USA) were cultured in DMEM supplemented with 10% FBS (Gibco, Grand Island, NY, USA).

### PPARγ ligand treatment

For *in vitro* PPARγ ligand treatment, 9-HODE (Cayman Chemical Co., Ann Arbor, MI, USA) was added into the culture medium of MDSCs to a final concentration of 20 μmol/L for 24 or 48 h. For the study of the effect of PPARγ ligand on the mTOR signaling pathway, bone marrow cells were treated with 9-HODE (20 μmol/L) for 2 h.

### Isolation of bone marrow-derived MDSCs

MDSCs were isolated as we previously described [5, 6]. Unlike those being classified into monocytic and granulocytic MDSCs, almost all *lal*^−/−^ MDSCs are Ly6G^+^Ly6C^+^, and almost all *lal*^−/−^ MDSCs are CD11b^+^Ly6G^+^ cells. Therefore, to simplify the *lal*^−/−^ MDSCs isolation procedure, Ly6G antibody-coupled magnetic beads were used and sufficient to isolate *lal*^−/−^ MDSCs from the *lal*^−/−^ bone marrow, and equivalent control from the wild type bone marrow [1, 2]. Briefly, bone marrow cells were isolated from the femurs and tibias of mice. Cells were first incubated with biotin-conjugated anti-Ly6G antibody at 4°C for 15 min. After washed with PBS, cells were incubated with anti-biotin microbeads at 4°C for another 15 min. Subsequently, cells were subjected to magnetic bead sorting according to the manufacturer's instructions (Miltenyi Biotec., Auburn, CA, USA).

### Mouse tumor growth and metastasis model

The tumor growth and metastasis model have been described recently [5]. MDSCs and B16 melanoma cells were collected separately. A pilot study has been performed to determine the best ratio between MDSCs and B16 melanoma cells. To test the tumor growth potential, 6 × 10^5^ pre-treated MDSCs and 2 × 10^5^ B16 melanoma cells were mixed, centrifuged and re-suspended in 100 μL PBS, and then injected subcutaneously into the flank region of 3-month old recipient *lal*^+/+^ mice. Tumor volume (length × width^2^ × π/6) was monitored every week for 4 weeks. To test the metastasis potential, 2 × 10^6^ pre-treated MDSCs and 5 × 10^5^ B16 melanoma cells were mixed and incubated at 37°C, 5% CO_2_ for 30 min. After the incubation, cells were centrifuged, re-suspended, and injected intravenously into 3-month old *lal*^+/+^ mice. Two weeks after the injection, the mice were sacrificed and the lungs were inflated with 4% paraformaldehyde for examination of metastasis.

### Histology and immunohistochemical staining

The harvested lungs were fixed with 4% paraformaldehyde in PBS at 4°C for overnight. After fixation and embedding in paraffin, tissue sections were cut to 5 μm thick. Hematoxylin and eosin (H&E) staining and immunohistochemical (IHC) staining with anti-Ki67 antibody were performed by the Histological Core Facility, Department of Pathology and Laboratory Medicine, Indiana University. Images were taken by Olympus microscopy image system (Olympus, Tokyo, Japan).

### *In vitro* co-culture of MDSCs and B16 melanoma cells

Previous study has determined the best ratio between MDSCs and B16 melanoma cells [5]. Ethanol or 20 μmol/L 9-HODE pre-treated (for 24 h) MDSCs (5 × 10^5^) and B16 melanoma cells (5 × 10^3^) were mixed, and seeded into a well of 96-well plates in DMEM supplemented with 10% FBS. Seventy-two hours later, unattached MDSCs were removed by washing with PBS, and the number of attached B16 melanoma cells was counted. Morphologically, MDSCs are much smaller than B16 melanoma cells for exclusion.

### *In vitro* migration assay

*In vitro* wound healing assay was performed to analyze B16 melanoma cell migration as previously described [16, 41]. Briefly, B16 melanoma cells were seeded at a density of 1.5 × 10^5^ cells/well into a 24-well plate and incubated overnight to form a confluent monolayer. Scratch was created by scraping the cell monolayer in a straight line with a p200 pipet tip. After washing 3 times with DMEM, the medium was changed with DMEM containing 10% FBS and 5 μg/mL mitomycin C (Sigma-Aldrich, St. Louis, MO, USA), and MDSCs pre-treated with 9-HODE or ethanol for 24 h were added onto B16 melanoma cell monolayer at a density of 1 × 10^6^ cells/well. Images were taken at 0 and 24 h after creating the scratch. Migration was estimated by measuring the distances from one side of scratch to the other side using Image Pro-Plus software (Media Cybernetics, Rockville, MD, USA).

### Transwell assay

Transwell assay was used to determine MDSC transendothelial migration [16]. SVECs were added to the upper chamber of 24-well 8.0-μm-pore Transwell plates (Corning, Corning, NY, USA), and incubated at 37°C, 5% CO_2_ for 48 h to form an EC monolayer. The supernatant was then removed, and CellTrackerTM Green 5-Chloromethylfluorescein Diacetate (CMFDA) (Invitrogen, Grand Island, NY, USA)-labeled MDSCs (2 × 10^4^ cells in 200 μL media) were added to the upper well. After 4 h, transendothelial migration of MDSCs was determined by counting their numbers in the lower chamber under 5 random microscopic fields.

To observe the effect of MDSCs-secreted cytokines on melanoma cell proliferation, transwell assay was performed with 0.4-μm-pore 6.5-mm diameter Transwell plates (Corning) to separate MDSCs and B16 melanoma cells. One million pre-treated MDSCs in 200 μL media were seeded into the upper chamber of the plates, while 2 × 10^4^ melanoma cells in 500 μL media were placed in the lower chamber. After 72 hours' culture, the transwells were removed, and the number of B16 melanoma cells in the lower chamber was counted.

### Isolation of bone marrow lineage-negative cells

Lineage-negative (Lin^−^) cells were isolated from the bone marrow by removing blood lineage marker-positive cells with an immunomagnetic microbead technique as we previously described [20]. Briefly, bone marrow cells were first incubated with a cocktail of biotin-conjugated antibodies against lineage specific antigens: CD11b, GR-1, B220, TER-119, and CD3ε (Mouse Lineage Panel Kit, BD Pharmingen, San Diego, CA, USA) at 4°C for 15 min. After washed with PBS, cells were then incubated with anti-biotin microbeads at 4°C for another 15 min. Subsequently, cells were subjected to magnetic bead sorting according to the manufacturer's instructions (Miltenyi Biotec.). The resulting Lin^−^ cells were cultured in RPMI1640 with 10% FBS. Five days later, Ly6G^+^CD11b^+^ cells derived from Lin^−^ cells were analyzed by flow cytometry analysis.

### ROS and mitochondrial membrane potential measurement

The reactive oxygen species (ROS) level and mitochondrial membrane potential in MDSCs was measured by flow cytometry as we previously described [20]. Briefly, bone marrow cells were first treated with or without 20 μmol/L 9-HODE or ethanol for 2 days. For ROS level detection, cells were harvested, washed, and stained with 2′, 7′-dichlorofluorescein diacetate (2 μmol/L, Invitrogen), allophycocyanin cy7-conjugated anti-Ly6G Ab, and phycoerythrin cy7-conjugated anti-CD11b Ab (eBioscience) at 37°C for 15 min. After PBS wash, the ROS level in Ly6G^+^CD11b^+^ cells was analyzed using a LSRII machine (BD Biosciences).

For mitochondrial membrane potential measurement, cells were stained with the fluorescent dye JC-1 (2 μmol/L, Molecular Probes, Eugene, OR, USA), allophycocyanin cy7-conjugated anti-Ly6G antibody, and phycoerythrin cy7-conjugated anti-CD11b antibody (eBioscience) at 37°C for 15 min, and then analyzed for phycoerythrin (JC-1 red) and fluorescein isothiocyanate (JC-1 green) fluorescent cells in Ly6G^+^CD11b^+^ cells by flow cytometry. Cells treated with 50 μmol/L carbonyl cyanide 3-chlorophenylhydrazone for 5 min were served as a fluorescein isothiocyanate-positive control.

### Flow cytometry analysis

Single cells from the bone marrow of 5-month-old *lal*^+/+^ and *lal*^−/−^ mice were prepared as previously described [9]. After 20 μmol/L 9-HODE treatment for 2 h, cells were harvested, and labeled with anti-Ly6G and CD11b cell surface antibody (eBioscience) at 4°C for 15 min. Cells were then fixed and permeabilized using BD Cytofix/Cytoperm Fixation/Permeabilization Kit (BD Biosciences) according to the manufacturer's instructions, and incubated with Alexa Fluor 647-conjugated anti-mTOR antibody, Alexa Fluor 488-conjugated anti-S6 antibody, Alexa Fluor 488-conjugated anti-pS6 (Ser235/236) antibody, and rabbit anti-pmTOR (Ser2448) antibody (Cell Signaling Technology, Beverly, MA, USA) at 4°C overnight. For anti-pmTOR antibody staining, cells were incubated with Alexa Fluor 647-conjugated anti-rabbit IgG in the following day. Cells were washed and ready for flow cytometry analysis. Mean fluorescence intensities of the proteins in the gated Ly6G^+^CD11b^+^ area were analyzed. For flow cytometry analysis, ≥ 10,000 cells were acquired and scored using a LSRII machine (BD Biosciences). Data were processed using the CellQuest software program (BD Biosciences).

### Statistics

Data were expressed as mean ± SD. Differences between two treatment groups were compared by Student's *t*-test. When more than two groups were compared, one-way ANOVA with post-hoc Newman-Keul's multiple comparison test was used. Results were considered statistically significant when *P* < 0.05. All analyses were performed with GraphPad Prism 5.0 (GraphPad, San Diego, CA, USA).
